# Supplementary material for: Some Specifics of Defect-Free Poly-(o-aminophenylene)naphthoylenimide Fibers Preparation by Wet Spinning
Source: Materials (Basel). 2022 Jan 21;15(3):808. doi: 10.3390/ma15030808 (PMC8836713; doi:10.3390/ma15030808)
Supplement: Supplementary file 1 [file materials-15-00808-s001.zip › materials-1542805-supplementary.pdf]

Supplementary Files

Some Specifics of Defect-Free Poly-(o aminophenylene)naphthoylenimide Fibers Preparation by Wet Spinning

Ivan Yu. Skvortsov [1]*, Valery G. Kulichikhin [1], Igor I. Ponomarev [2], Lydia A. Varfolomeeva [1], Mikhail S. Kuzin [1], Dmitry Y. Razorenov [2] and Kirill M. Skupov [2]

[1] A.V. Topchiev Institute of Petrochemical Synthesis of Russian Academy of Sciences, Leninsky Av., 29, Moscow 119991, Russia; klch@ips.ac.ru (V.G.K.); varfolomeeva.lidia@mail.ru (L.A.V.); gevahka15@gmail.com (M.S.K.)

[2] A.N. Nesmeyanov Institute of Organoelement Compounds of Russian Academy of Sciences, Vavilova St., 28, Moscow 119991, Russia; gagapon@ineos.ac.ru (I.I.P.); razar@ineos.ac.ru (D.Y.R.); kskupov@gmail.com (K.M.S.)

* Correspondence: amber5@yandex.ru

Coagulation with water:

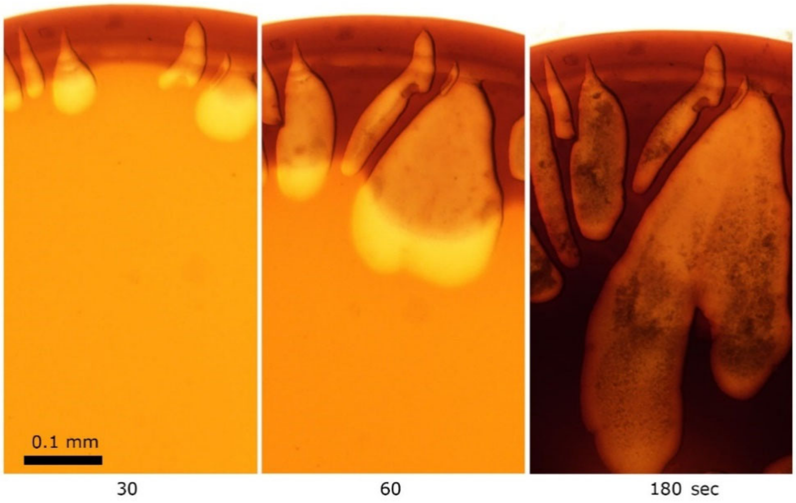

Figure S1. The coagulation kinetics of the PANI-O solution (12.7%, [η] = 3) under the action of water coagulant.

Another series of the coagulation of the high molecular weight solution by water.

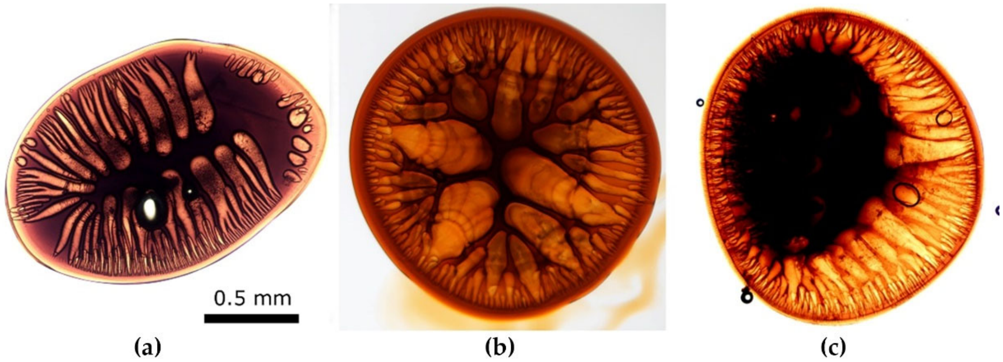

Figure S2. Water coagulated drops of PANI-O solutions: (**a**) 13%, [η] = 2.9; (**b**) 12.7%, [η] = 2.7; (**c**) 12% [η] = 1.8.

Some examples of coagulation with solvent/alcohols mixtures:

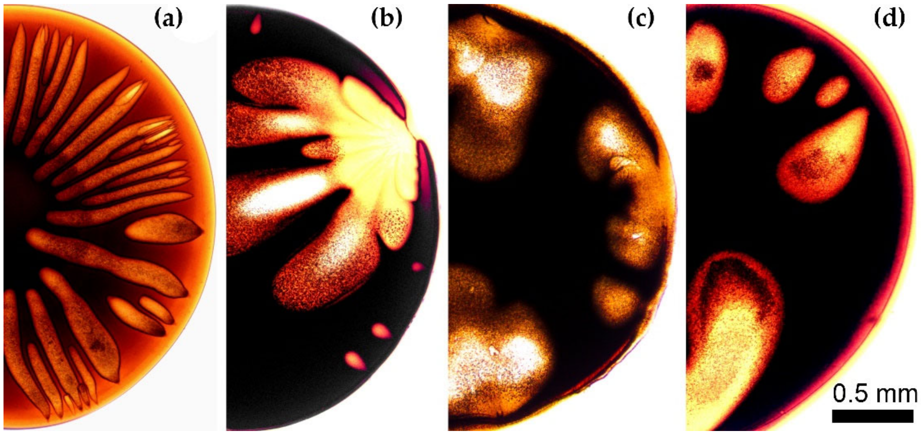

Figure S3. Image of P2 solution drop after contact with aliphatic alcohol/NMP (40/60 ratio) mixtures. (**a**)—ethanol, (**b**)—butanol-1, (**c**)—heptanol-1, (**d**)— octanol-1.

In the homologous series of alcohols, a slight decrease in the degree of defectiveness of the coagulated droplet starting with butanol is observed. In this case, the darkening area of the droplets increases along with an increase of the alcohol molecular weight, that means reinforcing the "diffusion coagulation" mode. Nevertheless, the morphology remains defective; therefore, such mixtures are not of interest from the viewpoint of PANI-O solutions coagulants.

Coagulation of P2 solution with alcohols of different viscosity and functionality:

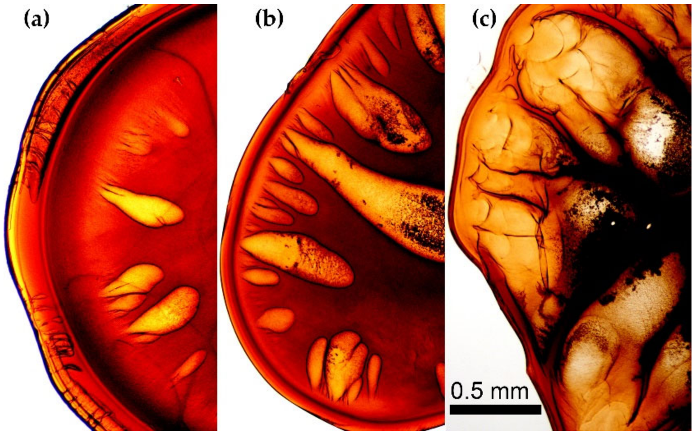

Figure S4. Coagulant: (**a**)—ethanol, (**b**)—ethylene glycol, (**c**)—glycerol.
